# Supplementary figures and images for: Mapping expanded prostate cancer index composite to EQ5D utilities to inform economic evaluations in prostate cancer: Secondary analysis of NRG/RTOG 0415
Source: PLoS One. 2021 Apr 14;16(4):e0249123. doi: 10.1371/journal.pone.0249123 (PMC8046237; doi:10.1371/journal.pone.0249123)

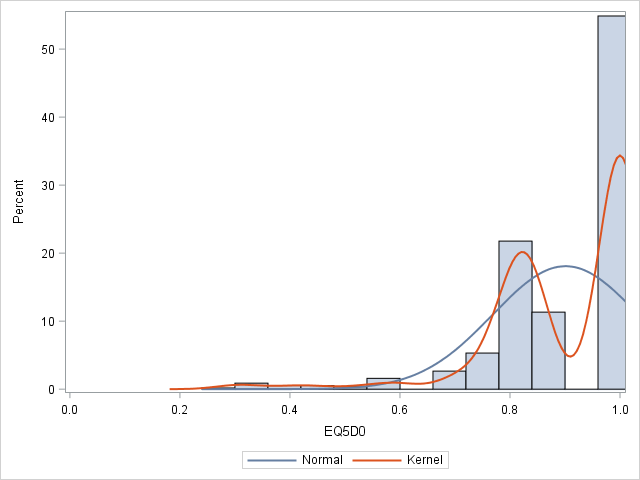

Supplement: S1 Fig — A. Patients with Complete Epic Domain Data (N = 565). B. Patients with Complete Epic Sub-Domain Data (N = 507). (ZIP) [file pone.0249123.s001.zip › S1A_Fig.tif]

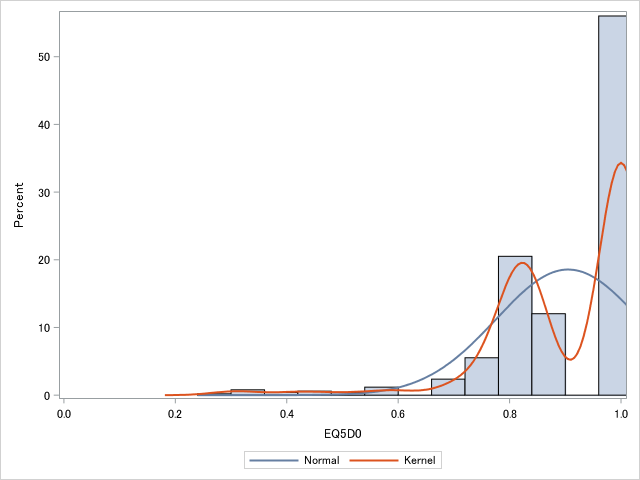

Supplement: S1 Fig — A. Patients with Complete Epic Domain Data (N = 565). B. Patients with Complete Epic Sub-Domain Data (N = 507). (ZIP) [file pone.0249123.s001.zip › S1B_Fig.tif]

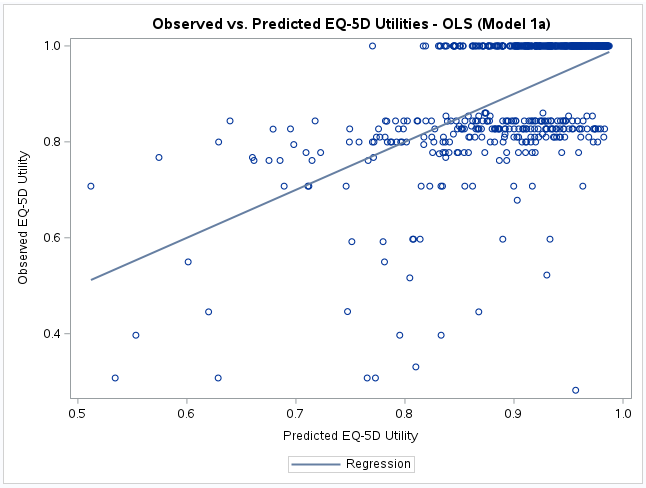

Supplement: S2 Fig — (ZIP) [file pone.0249123.s002.zip › S2A_Fig.tif]

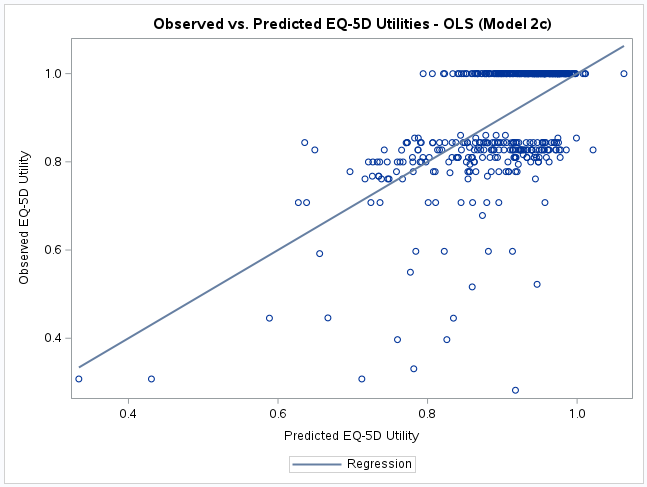

Supplement: S2 Fig — (ZIP) [file pone.0249123.s002.zip › S2B_Fig.tif]

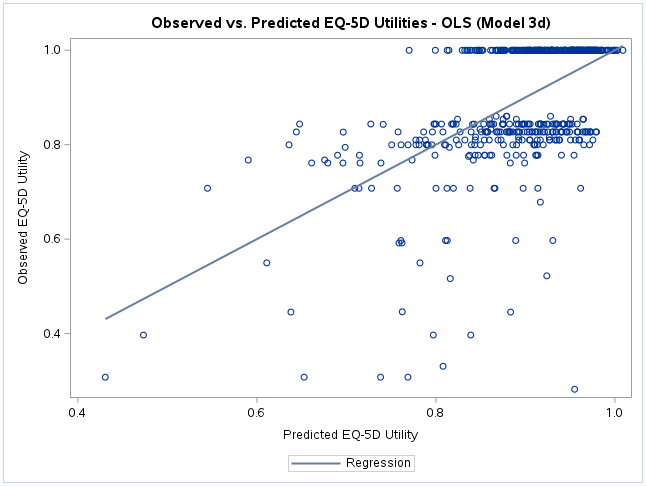

Supplement: S2 Fig — (ZIP) [file pone.0249123.s002.zip › S2C_Fig.tif]

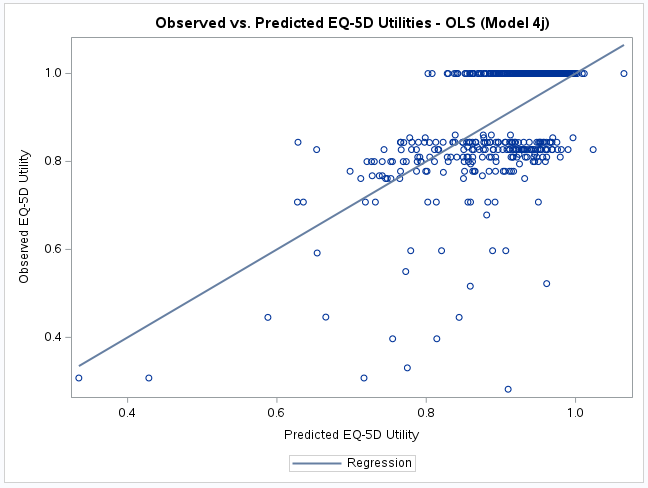

Supplement: S2 Fig — (ZIP) [file pone.0249123.s002.zip › S2D_Fig.tif]

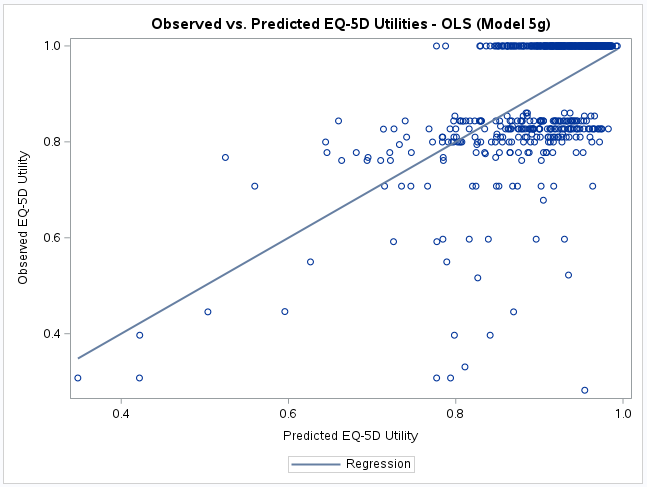

Supplement: S2 Fig — (ZIP) [file pone.0249123.s002.zip › S2E_Fig.tif]

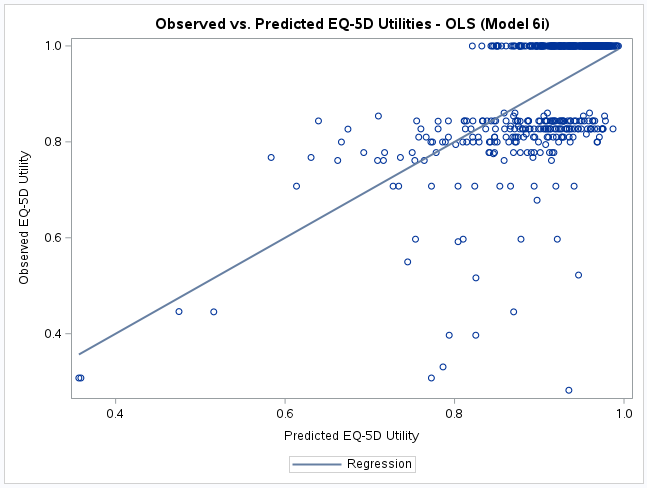

Supplement: S2 Fig — (ZIP) [file pone.0249123.s002.zip › S2F_Fig.tif]

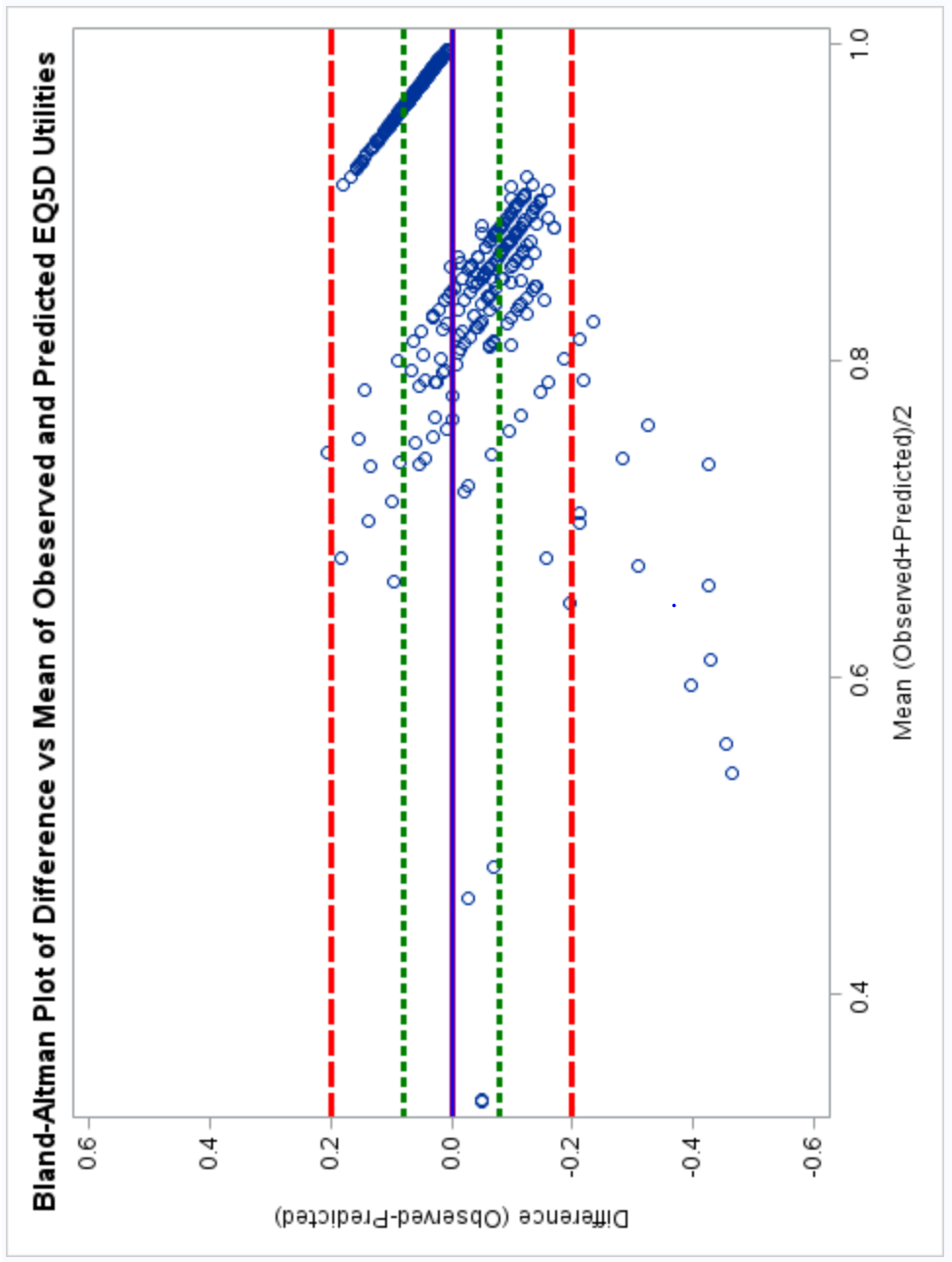

Supplement: S3 Fig — (TIF) [file pone.0249123.s003.tif]
