# Supplementary material for: Mapping expanded prostate cancer index composite to EQ5D utilities to inform economic evaluations in prostate cancer: Secondary analysis of NRG/RTOG 0415
Source: PLoS One. 2021 Apr 14;16(4):e0249123. doi: 10.1371/journal.pone.0249123 (PMC8046237; doi:10.1371/journal.pone.0249123)
Supplement: S1 Table — (DOCX) [file pone.0249123.s004.docx]

| **S1 Table: Model Specifications** | | | |
| --- | --- | --- | --- |
| Group | Available Data | Model ID | Explanatory Variables |
| 1 | EPIC Domains | 1a | U, B S, H |
|  |  | 1b | U, B S, H, U^2^, B^2^, S^2^, H^2^ |
|  |  | 1c | U, B S, H, U^2^, B^2^, S^2^, H^2^, U^3^, B^3^, S^3^, H^3^ |
| 2 | EPIC Sub-Domains | 2a | UB, BB, SB, HB, UF, BF, SF, HF, UI, UIC |
|  |  | 2b | UB, BB, SB, HB, UF, BF, SF, HF, UI, UIC, UB^2^, BB^2^, SB^2^, HB^2^, UF^2^, BF^2^, SF^2^, HF^2^, UI^2^, UIC^2^ |
|  |  | 2c | UB, BB, SB, HB, UF, BF, SF, HF, UI, UIC, UB^2^, BB^2^, SB^2^, HB^2^, UF^2^, BF^2^, SF^2^, HF^2^, UI^2^, UIC^2^, UB^3^, BB^3^, SB^3^, HB^3^, UF^3^, BF^3^, SF^3^, HF^3^, UI^3^, UIC^3^ |
| 3 | EPIC Domains, Age, Race | 3a | U, B S, H, Age, Race |
|  |  | 3b | U, B S, H, Age, Age^2^, Race |
|  |  | 3c | U, B S, H, Age, Age^2^, Age^3^, Race |
|  |  | 3d | U, B S, H, Age, Race, U* Race, B* Race, S* Race, H* Race |
|  |  | 3e | U, B S, H, Age, Age^2^, Race, U* Race, B* Race, S* Race, H* Race |
|  |  | 3f | U, B S, H, Age, Age^2^, Age^3^, Race, U* Race, B* Race, S* Race, H* Race |
| 4 | EPIC Sub-Domains, Age, Race | 4a | UB, BB, SB, HB, UF, BF, SF, HF, UI, UIC, Age, Race |
|  |  | 4b | UB, BB, SB, HB, UF, BF, SF, HF, UI, UIC, Age, Age^2^, Race |
|  |  | 4c | UB, BB, SB, HB, UF, BF, SF, HF, UI, UIC, Age, Age^2^, Age^3^, Race |
|  |  | 4d | UB, BB, SB, HB, UF, BF, SF, HF, UI, UIC, Age, Race, UB * Race, BB * Race, SB * Race, HB * Race, UF * Race, BF * Race, SF * Race, HF * Race, UI * Race, UIC * Race |
|  |  | 4e | UB, BB, SB, HB, UF, BF, SF, HF, UI, UIC, Age, Age^2^, Race, UB * Race, BB * Race, SB * Race, HB * Race, UF * Race, BF * Race, SF * Race, HF * Race, UI * Race, UIC * Race |
|  |  | 4f | UB, BB, SB, HB, UF, BF, SF, HF, UI, UIC, Age, Age^2^, Age^3^, Race, UB * Race, BB * Race, SB * Race, HB * Race, UF * Race, BF * Race, SF * Race, HF * Race, UI * Race, UIC * Race |
|  |  | 4g | UB, BB, SB, HB, UF, BF, SF, HF, UI, UIC, UB^2^, BB^2^, SB^2^, HB^2^, UF^2^, BF^2^, SF^2^, HF^2^, UI^2^, UIC^2^, Age, Race |
|  |  | 4h | UB, BB, SB, HB, UF, BF, SF, HF, UI, UIC, UB^2^, BB^2^, SB^2^, HB^2^, UF^2^, BF^2^, SF^2^, HF^2^, UI^2^, UIC^2^, Age, Age^2^, Race |
|  |  | 4i | UB, BB, SB, HB, UF, BF, SF, HF, UI, UIC, UB^2^, BB^2^, SB^2^, HB^2^, UF^2^, BF^2^, SF^2^, HF^2^, UI^2^, UIC^2^, Age, Age^2^, Age^3^, Race |
|  |  | 4j | UB, BB, SB, HB, UF, BF, SF, HF, UI, UIC, UB^2^, BB^2^, SB^2^, HB^2^, UF^2^, BF^2^, SF^2^, HF^2^, UI^2^, UIC^2^, Age, Race |
|  |  | 4k | UB, BB, SB, HB, UF, BF, SF, HF, UI, UIC, UB^2^, BB^2^, SB^2^, HB^2^, UF^2^, BF^2^, SF^2^, HF^2^, UI^2^, UIC^2^, UB^3^, BB^3^, SB^3^, HB^3^, UF^3^, BF^3^, SF^3^, HF^3^, UI^3^, UIC^3^, Age, Age^2^, Race |
|  |  | 4l | UB, BB, SB, HB, UF, BF, SF, HF, UI, UIC, UB^2^, BB^2^, SB^2^, HB^2^, UF^2^, BF^2^, SF^2^, HF^2^, UI^2^, UIC^2^, UB^3^, BB^3^, SB^3^, HB^3^, UF^3^, BF^3^, SF^3^, HF^3^, UI^3^, UIC^3^, Age, Age^2^, Age^3^, Race |
| 5 | EPIC Domains, Age, Race, Zubrod, PSA | 5a | U, B S, H, Age, Race, Zubrod, PSA |
|  |  | 5b | U, B S, H, Age, Age^2^, Race, Zubrod, PSA |
|  |  | 5c | U, B S, H, Age, Age^2^, Age^3^, Race, Zubrod, PSA |
|  |  | 5d | U, B S, H, Age, Race, U* Race, B* Race, S* Race, H* Race, Zubrod, PSA |
|  |  | 5e | U, B S, H, Age, Age^2^, Race, U* Race, B* Race, S* Race, H* Race, Zubrod, PSA |
|  |  | 5f | U, B S, H, Age, Age^2^, Age^3^, Race, U* Race, B* Race, S* Race, H* Race, Zubrod, PSA |
|  |  | 5g | U, B, S, H, Age, Race, Zubrod, U* Zubrod, B* Zubrod, S* Zubrod, H* Zubrod, PSA |
|  |  | 5h | U, B, S, H, Age, Age^2^, Race, Zubrod, U* Zubrod, B* Zubrod, S* Zubrod, H* Zubrod, PSA |
|  |  | 5i | U, B, S, H, Age, Age^2^, Age^3^, Race, Zubrod, U* Zubrod, B* Zubrod, S* Zubrod, H* Zubrod, PSA |
|  |  | 5j | U, B S, H, Age, Race, U* Race, B* Race, S* Race, H* Race, Zubrod, U* Zubrod, B* Zubrod, S* Zubrod, H* Zubrod, PSA |
|  |  | 5k | U, B S, H, Age, Age^2^, Race, U* Race, B* Race, S* Race, H* Race, Zubrod, U* Zubrod, B* Zubrod, S* Zubrod, H* Zubrod, PSA |
|  |  | 5l | U, B S, H, Age, Age^2^, Age^3^, Race, U* Race, B* Race, S* Race, H* Race, Zubrod, U* Zubrod, B* Zubrod, S* Zubrod, H* Zubrod, PSA |
| 6 | EPIC Sub-Domains, Age, Race, Zubrod, PSA | 6a | UB, BB, SB, HB, UF, BF, SF, HF, UI, UIC, Age, Race, Zubrod, PSA |
|  |  | 6b | UB, BB, SB, HB, UF, BF, SF, HF, UI, UIC, Age, Age^2^, Race, Zubrod, PSA |
|  |  | 6c | UB, BB, SB, HB, UF, BF, SF, HF, UI, UIC, Age, Age^2^, Age^3^, Race, Zubrod, PSA |
|  |  | 6d | UB, BB, SB, HB, UF, BF, SF, HF, UI, UIC, Age, Race, Race, UB * Race, BB * Race, SB * Race, HB * Race, UF * Race, BF * Race, SF * Race, HF * Race, UI * Race, UIC * Race, Zubrod, PSA |
|  |  | 6e | UB, BB, SB, HB, UF, BF, SF, HF, UI, UIC, Age, Age^2^, Race, UB * Race, BB * Race, SB * Race, HB * Race, UF * Race, BF * Race, SF * Race, HF * Race, UI * Race, UIC * Race, Zubrod, PSA |
|  |  | 6f | UB, BB, SB, HB, UF, BF, SF, HF, UI, UIC, Age, Age^2^, Age^3^, Race, UB * Race, BB * Race, SB * Race, HB * Race, UF * Race, BF * Race, SF * Race, HF * Race, UI * Race, UIC * Race, Zubrod, PSA |
|  |  | 6g | UB, BB, SB, HB, UF, BF, SF, HF, UI, UIC, Age, Race, Zubrod, UB* Zubrod, BB* Zubrod, SB* Zubrod, HB* Zubrod, UF* Zubrod, BF* Zubrod, SF* Zubrod, HF* Zubrod, UI* Zubrod, UIC* Zubrod, PSA |
|  |  | 6h | UB, BB, SB, HB, UF, BF, SF, HF, UI, UIC, Age, Age^2^, Race, Zubrod, UB* Zubrod, BB* Zubrod, SB* Zubrod, HB* Zubrod, UF* Zubrod, BF* Zubrod, SF* Zubrod, HF* Zubrod, UI* Zubrod, UIC* Zubrod, PSA |
|  |  | 6i | UB, BB, SB, HB, UF, BF, SF, HF, UI, UIC, Age, Age^2^, Age^3^, Race, Zubrod, UB* Zubrod, BB* Zubrod, SB* Zubrod, HB* Zubrod, UF* Zubrod, BF* Zubrod, SF* Zubrod, HF* Zubrod, UI* Zubrod, UIC* Zubrod, PSA |
|  |  | 6j | UB, BB, SB, HB, UF, BF, SF, HF, UI, UIC, Age, Race, Race, UB * Race, BB * Race, SB * Race, HB * Race, UF * Race, BF * Race, SF * Race, HF * Race, UI * Race, UIC * Race, Zubrod, UB* Zubrod, BB* Zubrod, SB* Zubrod, HB* Zubrod, UF* Zubrod, BF* Zubrod, SF* Zubrod, HF* Zubrod, UI* Zubrod, UIC* Zubrod, PSA |
|  |  | 6k | UB, BB, SB, HB, UF, BF, SF, HF, UI, UIC, Age, Age^2^, Race, UB * Race, BB * Race, SB * Race, HB * Race, UF * Race, BF * Race, SF * Race, HF * Race, UI * Race, UIC * Race, Zubrod, UB* Zubrod, BB* Zubrod, SB* Zubrod, HB* Zubrod, UF* Zubrod, BF* Zubrod, SF* Zubrod, HF* Zubrod, UI* Zubrod, UIC* Zubrod, PSA |
|  |  | 6l | UB, BB, SB, HB, UF, BF, SF, HF, UI, UIC, Age, Age^2^, Age^3^, Race, UB * Race, BB * Race, SB * Race, HB * Race, UF * Race, BF * Race, SF * Race, HF * Race, UI * Race, UIC * Race, Zubrod, UB* Zubrod, BB* Zubrod, SB* Zubrod, HB* Zubrod, UF* Zubrod, BF* Zubrod, SF* Zubrod, HF* Zubrod, UI* Zubrod, UIC* Zubrod, PSA |

Abbreviations: U=Urinary Domain, B=Bowel Domain, S=Sexual Domain, H=Hormonal Domain, UF=Urinary Function, UB = Urinary Bother, UI= Urinary Irritation UIC=Urinary Incontinence, BF=Bowel Function, BB=Bowel Bother, SF=Sexual Function, SB=Sexual Bother, HF=Hormonal Function, HB=Hormonal Bother
