# Supplementary material for: Mapping expanded prostate cancer index composite to EQ5D utilities to inform economic evaluations in prostate cancer: Secondary analysis of NRG/RTOG 0415
Source: PLoS One. 2021 Apr 14;16(4):e0249123. doi: 10.1371/journal.pone.0249123 (PMC8046237; doi:10.1371/journal.pone.0249123)
Supplement: S2 Table — (DOCX) [file pone.0249123.s005.docx]

| **S2 Table:  Candidate Mapping Algorithms and External Validation Results in The 30% Sample RTOG 0415 Secondary Analysis** | | | | | | | |
| --- | --- | --- | --- | --- | --- | --- | --- |
|  |  |  | |  | Predicted EQ5D | |  |
| Model | Available Data | |  | Candidate Mapping Algorithm | Mean (SD) | Min-Max | RMSE |
|  | | | | | | | |
| Model 1A | EPIC Domain Scores | |  | Predicted EQ5D = 0.184907 + 0.002172*U + 0.005518*H + 0.000173*S + 0.000166*B | 0.90 (0.08) | 0.58 - 0.99 | 0.122668 |
|  | | | | | | | |
| Model 3D | EPIC Domain Scores, Age, Race | |  | Predicted EQ5D = 0.257467 + 0.001885*U + 0.005492*H + 0.000193*S – 0.000133*B – 0.271300*Race(Other) + 0.001465*U*Race(Other) – 0.000056009*H*Race(Other) – 0.000350*S*Race(Other) +0.001770*B*Race(Other) | 0.91 (0.08) | 0.61 - 1.02 | 0.124491 |
|  | | | | | | | |
| Model 5G | EPIC Domain Scores, Age, Race, Zubrod, PSA | |  | Predicted EQ5D = 0.290851 + 0.001738*U + 0.005315*H + 0.000284*S – 0.000387*B – 0.000050962*Age – 0.000077975*Race(Other) -0.446705*Zubrod(1) + 0.002327*U*Zubrod(1) – 0.002765*H*Zubrod(1)-0.001551*S*Zubrod(1) + 0.005463*B*Zubrod(1) + 0.005275*PSA(1) | 0.89 (0.08) | 0.56 - 0.99 | 0.122175 |
|  | | | | | | | |
| Model 2C | EPIC Sub-Domain Scores | |  | Predicted EQ5D =-5.047077 – 0.056428*UF – 0.001444*UB – 0.006706*UI +0.004702*UIC + 0.198935*BF + 0.081753*BB - 0.001678*SF + 0.000103*SB + 0.001138*HF – 0.028427*HB + 0.000887*UF^2^ + 0.000060029*UB^2^ + 0.000116*UI^2^ – 0.000152*UIC^2^ – 0.002375*BF^2^ – 0.001057*BB^2^ + 0.000047665*SF^2^ + 0.000008399*SB^2^ – 0.000007066*HF^2^ + 0.000364*HB^2^ – 0.000004090*UF^3^ – 0.000000125*UB^3^ – 0.000000857 *UI^3^ + 0.000000860*UIC^3^ + 0.000009347*BF^3^ + 0.000004437*BB^3^ – 0.000000289*SF^3^ – 0.000000116*SB^3^ + 0.000000003*HF^3^ – 0.000001278*HB^3^ | 0.89 (0.09) | 0.44 – 1.00 | 0.113311 |
|  | | | | | | | |
| Model 6I | EPIC Sub-Domain Scores, Age, Race, Zubrod, PSA | |  | Predicted EQ5D = 2.922434 + 0.003627*UF+ 0.004125*UB – 0.003625*UI – 0.002242*UIC – 0.0000058476*BF – 0.000690*BB + 0.000589*SF – 0.000244*SB + 0.000721*HF + 0.004691*HB – 0.126445*Age + 0.001997*Age^2^ – 0.000010336*Age^3^ + 0.009922*Race(Other) – 0.456669*Zubrod(1) + 0.016593*UF*Zubrod (1) + 0.008613*UB*Zubrod(1) – 0.011*UI*Zubrod(1) – 0.011342*UIC*Zubrod(1) + 0.000711*BF*Zubrod(1) + 0.003675*BB*Zubrod(1) – 0.001631*SF*Zubrod(1) + 0.00008517*SB*Zubrod – 0.000201*HF*Zubrod(1) – 0.002221*HB*Zubrod(1) + 0.000332*PSA(1) | 0.91 (0.07) | 0.67 - 1.01 | 0.110482 |
|  | | | | | | | |
| Stepwise Selection: Model 1/Model 3 | EPIC Domain Scores (with or without demographic covariates) | |  | Predicted EQ5D = 0.188704 + 0.002301*U + 0.005617*H | 0.90 (0.08) | 0.58 - 0.98 | 0.123367 |
|  | | | | | | | |
| Stepwise Selection: Model 5 | EPIC Domain Scores, Age, Race, Zubrod, PSA | |  | Predicted EQ5D = 0.268256 + 0.001777* U + 0.005292*H – 0.315614*Zubrod(1) + 0.003143*U*Zubrod(1) | 0.90 (0.08) | 0.51 - 0.98 | 0.123662 |
|  | | | | | | | |
| Stepwise Selection: Model 2/ Model 4 | EPIC Sub-Domain Scores (with or without demographic covariates) | |  | Predicted EQ5D = 0.158553 + 0.001364*UF + 0.001193*UB + 0.000998*HF + 0.004626*HB | 0.90 (0.07) | 0.65 - 0.98 | 0.113095 |
|  | | | | | | | |
| Stepwise Selection: Model 6 | EPIC Sub-domain Scores, Age, Race, Zubrod, PSA | |  | Predicted EQ5D = 0.248541 + 0.000748*UF + 0.001134*UB + 0.000968*HF + 0.004404*HB – 0.376487*Zubrod(1) + 0.003562*UF*Zubrod(1) | 0.90 (0.08) | 0.5 - 0.97 | 0.114714 |
|  | | | | | | | |
| Abbreviations: U=Urinary Domain, B=Bowel Domain, S=Sexual Domain, H=Hormonal Domain, UF=Urinary Function, UB=Urinary Bother, UI=Urinary Irritation UIC=Urinary Incontinence, BF=Bowel Function, BB=Bowel Bother, SF=Sexual Function, SB=Sexual Bother, HF=Hormonal Function, HB=Hormonal Bother | | | | | | | |
