# Supplementary material for: Mapping expanded prostate cancer index composite to EQ5D utilities to inform economic evaluations in prostate cancer: Secondary analysis of NRG/RTOG 0415
Source: PLoS One. 2021 Apr 14;16(4):e0249123. doi: 10.1371/journal.pone.0249123 (PMC8046237; doi:10.1371/journal.pone.0249123)
Supplement: S3 Table — (DOCX) [file pone.0249123.s006.docx]

| **S3 Table: Baseline Characteristics of Patients with Complete EPIC Domain Data** | | | |
| --- | --- | --- | --- |
|  | 30% Cohort (n=232) | 70% Cohort (n=565) | P-value* |
|  | | |  |
| Age |  |  | 0.917 |
| Mean | 66.2 | 66.4 |  |
| Std. Dev. | 7.7 | 7.3 |  |
| Median | 66 | 67 |  |
| Min - Max | 44 - 82 | 42 - 84 |  |
| Q1 - Q3 | 61.5 - 72 | 62 - 72 |  |
|  | | |  |
| Baseline PSA |  |  | 0.759 |
| <4 | 45 ( 19.4%) | 115 ( 20.4%) |  |
| ≥4 | 187 ( 80.6%) | 450 ( 79.6%) |  |
|  |  |  |  |
| Mean | 5.5 | 5.6 |  |
| Std. Dev. | 2.2 | 2.1 |  |
| Median | 5.2 | 5.47 |  |
| Min - Max | 0.1 - 9.92 | 0.33 - 9.98 |  |
| Q1 - Q3 | 4.28 - 7.035 | 4.17 - 6.92 |  |
|  | | |  |
| Race |  |  | 0.082 |
| Other | 53 ( 22.8%) | 99 ( 17.5%) |  |
| White | 179 ( 77.2%) | 466 ( 82.5%) |  |
|  | | |  |
| Zubrod |  |  | 0.152 |
| 0 | 211 ( 90.9%) | 530 ( 93.8%) |  |
| 1 | 21 ( 9.1%) | 35 ( 6.2%) |  |
|  | | |  |
| *Chi-square test for categorical variables; Wilcoxon rank sum test for continuous variables | | | |
