# Supplementary material for: Mapping expanded prostate cancer index composite to EQ5D utilities to inform economic evaluations in prostate cancer: Secondary analysis of NRG/RTOG 0415
Source: PLoS One. 2021 Apr 14;16(4):e0249123. doi: 10.1371/journal.pone.0249123 (PMC8046237; doi:10.1371/journal.pone.0249123)
Supplement: S4 Table — (DOCX) [file pone.0249123.s007.docx]

| **S4 Table: EQ5D and EPIC Domain Scores for Patients with Complete EPIC Domain Data** | | | |
| --- | --- | --- | --- |
|  | 30% Cohort (n=232) | 70% Cohort (n=565) | P-value* |
|  | | |  |
| EQ5D |  |  |  |
| 1 | 120 (51.7) | 310 (54.9) |  |
| <1 | 112 (48.3) | 255 (45.1) |  |
|  |  |  |  |
| Mean | 0.89 | 0.90 | 0.554 |
| Std. Dev. | 0.14 | 0.13 |  |
| Median | 1.00 | 1.00 |  |
| Min - Max | 0.17 - 1.00 | 0.28 - 1.00 |  |
| Q1 - Q3 | 0.83 - 1.00 | 0.83 - 1.00 |  |
|  | | |  |
| EPIC Urinary Domain |  |  | 0.300 |
| Mean | 86.52 | 87.53 |  |
| Std. Dev. | 12.53 | 12.09 |  |
| Median | 91.67 | 91.00 |  |
| Min - Max | 29.17 - 100.00 | 29.83 - 100.00 |  |
| Q1 - Q3 | 78.50 - 95.83 | 81.25 - 97.92 |  |
|  | | |  |
| EPIC Bowel Domain |  |  | 0.056 |
| Mean | 92.71 | 93.43 |  |
| Std. Dev. | 9.23 | 9.26 |  |
| Median | 96.43 | 96.43 |  |
| Min - Max | 46.15 - 100.00 | 30.77 - 100.00 |  |
| Q1 - Q3 | 89.29 - 98.21 | 91.07 - 100.00 |  |
|  | | |  |
| EPIC Sexual Domain |  |  | 0.728 |
| Mean | 50.39 | 49.64 |  |
| Std. Dev. | 26.61 | 26.29 |  |
| Median | 52.75 | 51.55 |  |
| Min - Max | 0.00 - 97.92 | 0.00 - 98.08 |  |
| Q1 - Q3 | 29.81 - 73.08 | 28.85 - 71.15 |  |
|  | | |  |
| EPIC Hormonal Domain |  |  |  |
| Mean | 90.47 | 90.96 | 0.986 |
| Std. Dev. | 11.75 | 11.03 |  |
| Median | 95.45 | 94.44 |  |
| Min - Max | 45.45 - 100.00 | 40.91 - 100.00 |  |
| Q1 - Q3 | 85.68 - 100.00 | 86.36 - 100.00 |  |
|  | | |  |
|  | | | |
