# Supplementary material for: Mapping expanded prostate cancer index composite to EQ5D utilities to inform economic evaluations in prostate cancer: Secondary analysis of NRG/RTOG 0415
Source: PLoS One. 2021 Apr 14;16(4):e0249123. doi: 10.1371/journal.pone.0249123 (PMC8046237; doi:10.1371/journal.pone.0249123)
Supplement: S5 Table — (DOCX) [file pone.0249123.s008.docx]

| **S5 Table: EPIC Sub-Domain Scores for Patients with Complete EPIC Sub-Domain Data** | | | |
| --- | --- | --- | --- |
|  | 30% Cohort (n=213) | 70% Cohort (n=507) | P-value* |
|  | | |  |
| EQ5D |  |  |  |
| 1 | 114 (53.5) | 284 (56.0) |  |
| <1 | 99 (46.5) | 223 (44.0) |  |
|  |  |  |  |
| Mean | 0.90 | 0.91 | 0.686 |
| Std. Dev. | 0.13 | 0.13 |  |
| Median | 1.00 | 1.00 |  |
| Min - Max | 0.31-1.00 | 0.28-1.00 |  |
| Q1 - Q3 | 0.83-1.00 | 0.83-1.00 |  |
|  |  |  |  |
| Urinary Function |  |  | 0.908 |
| Mean | 92.92 | 93.28 |  |
| Std. Dev. | 11.81 | 10.74 |  |
| Median | 100.00 | 100.00 |  |
| Min - Max | 40.00 - 100.00 | 31.60 - 100.00 |  |
| Q1 - Q3 | 90.00 - 100.00 | 90.00 - 100.00 |  |
|  | | |  |
| Urinary Bother |  |  | 0.164 |
| Mean | 82.70 | 84.04 |  |
| Std. Dev. | 14.84 | 14.83 |  |
| Median | 89.29 | 87.50 |  |
| Min - Max | 21.43 - 100.00 | 17.86 - 100.00 |  |
| Q1 - Q3 | 71.43 - 92.86 | 75.00 - 96.43 |  |
|  | | |  |
| Urinary Irritation |  |  | 0.097 |
| Mean | 85.61 | 86.80 |  |
| Std. Dev. | 12.07 | 12.61 |  |
| Median | 89.29 | 89.29 |  |
| Min - Max | 35.71 - 100.00 | 25.00 - 100.00 |  |
| Q1 - Q3 | 78.57 - 92.86 | 82.14 - 96.43 |  |
|  | | |  |
| Urinary Incontinence |  |  | 0.916 |
| Mean | 91.26 | 91.61 |  |
| Std. Dev. | 14.84 | 14.01 |  |
| Median | 100.00 | 100.00 |  |
| Min - Max | 25.00 - 100.00 | 14.50 - 100.00 |  |
| Q1 - Q3 | 85.50 - 100.00 | 85.50 - 100.00 |  |
|  | | |  |
| Bowel Function |  |  | 0.179 |
| Mean | 92.35 | 93.21 |  |
| Std. Dev. | 9.41 | 8.49 |  |
| Median | 96.43 | 96.43 |  |
| Min - Max | 53.57 - 100.00 | 53.57 - 100.00 |  |
| Q1 - Q3 | 89.29 - 100.00 | 89.29 - 100.00 |  |
|  | | |  |
| Bowel Bother |  |  | 0.090 |
| Mean | 93.41 | 94.58 |  |
| Std. Dev. | 10.72 | 9.56 |  |
| Median | 96.43 | 100.00 |  |
| Min - Max | 32.14 - 100.00 | 28.57 - 100.00 |  |
| Q1 - Q3 | 89.29 - 100.00 | 92.86 - 100.00 |  |
|  | | |  |
| Sexual Function |  |  | 0.500 |
| Mean | 45.11 | 43.66 |  |
| Std. Dev. | 27.46 | 26.91 |  |
| Median | 49.11 | 48.11 |  |
| Min - Max | 0.00 - 96.88 | 0.00 - 97.22 |  |
| Q1 - Q3 | 23.11 - 66.67 | 18.56 - 66.67 |  |
|  | | |  |
| Sexual Bother |  |  | 0.728 |
| Mean | 64.88 | 64.02 |  |
| Std. Dev. | 32.39 | 32.91 |  |
| Median | 68.75 | 68.75 |  |
| Min - Max | 0.00 - 100.00 | 0.00 - 100.00 |  |
| Q1 - Q3 | 43.75 - 100.00 | 37.50 - 100.00 |  |
|  | | |  |
| Hormonal Function |  |  | 0.707 |
| Mean | 88.71 | 88.68 |  |
| Std. Dev. | 13.52 | 13.64 |  |
| Median | 95.00 | 90.00 |  |
| Min - Max | 40.00 - 100.00 | 10.00 - 100.00 |  |
| Q1 - Q3 | 80.00 - 100.00 | 85.00 - 100.00 |  |
|  | | |  |
| Hormonal Bother |  |  | 0.684 |
| Mean | 92.59 | 93.05 |  |
| Std. Dev. | 10.32 | 10.32 |  |
| Median | 95.83 | 95.83 |  |
| Min - Max | 50.00 - 100.00 | 25.00 - 100.00 |  |
| Q1 - Q3 | 87.50 - 100.00 | 91.67 - 100.00 |  |
|  | | |  |
| *Wilcoxon rank sum test | | | |
