# Supplementary material for: Mapping expanded prostate cancer index composite to EQ5D utilities to inform economic evaluations in prostate cancer: Secondary analysis of NRG/RTOG 0415
Source: PLoS One. 2021 Apr 14;16(4):e0249123. doi: 10.1371/journal.pone.0249123 (PMC8046237; doi:10.1371/journal.pone.0249123)
Supplement: S6 Table — (DOCX) [file pone.0249123.s009.docx]

| **S6 Table: Baseline Characteristics of Patients Included vs Not Included in Complete EPIC Sub-Domain Analysis** | | | |
| --- | --- | --- | --- |
|  | Excluded from EPIC Sub-Domain Analysis (n=58) | Included in EPIC Sub-Domain Analysis (n=507) | P-value* |
|  | | |  |
| Age |  |  | 0.825 |
| Mean | 66.4 | 66.4 |  |
| Std. Dev. | 8.3 | 7.2 |  |
| Median | 67 | 66 |  |
| Min - Max | 49 - 82 | 42 - 84 |  |
| Q1 - Q3 | 62 - 72 | 62 - 72 |  |
|  | | |  |
| Baseline PSA |  |  | 0.782 |
| <4 | 11 ( 19.0%) | 104 ( 20.5%) |  |
| ≥4 | 47 ( 81.0%) | 403 ( 79.5%) |  |
|  |  |  |  |
| Mean | 5.7 | 5.6 |  |
| Std. Dev. | 2.2 | 2.1 |  |
| Median | 5.79 | 5.42 |  |
| Min - Max | 0.33 - 9.82 | 0.47 - 9.98 |  |
| Q1 - Q3 | 4.1 - 7.34 | 4.17 - 6.92 |  |
|  | | |  |
| Race |  |  | 0.301 |
| Other | 13 ( 22.4%) | 86 ( 17.0%) |  |
| White | 45 ( 77.6%) | 421 ( 83.0%) |  |
|  | | |  |
| Zubrod |  |  | 0.419 |
| 0 | 53 ( 91.4%) | 477 ( 94.1%) |  |
| 1 | 5 ( 8.6%) | 30 ( 5.9%) |  |
|  | | |  |
| *Chi-square test for categorical variables; Wilcoxon rank sum test for continuous variables | | | |
